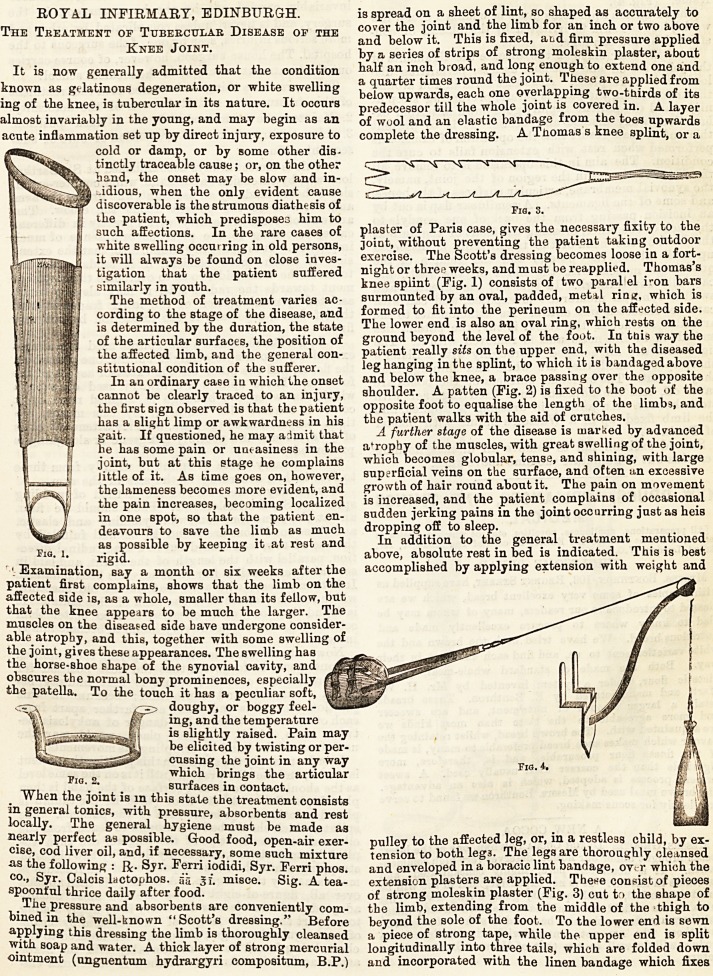# The Treatment of Tubercular Disease of the Knee-Joint

**Published:** 1892-11-26

**Authors:** 


					Nov. 26, 1892. THE HOSPITAL. 137
The Hospital Clinic.
[The Editor will be glad to receive offers of co-operation and contributions from members of the profession. All letters should be
addressed to The Editor, The Lodge, Porchester Square, London, W.]
ROYAL INFIRMARY, EDINBURGH.
The Treatment of Tubercular Disease of the
Knee Joint.
It is now generally admitted that the condition
known as gelatinous degeneration, or white swelling
ing of the knee, is tubercular in its nature. It occurs
almost invariably in the young, and may begin as an
acute inflammation set up by direct injury, exposure to
cold or damp, or by some other dis-
tinctly traceable cause; or, on the other
band, the onset may be slow and in-
sidious, when the only evident cause
discoverable is the strumous diathesis of
the patient, which predisposes him to
such affections. In the rare cases of
white swelling occmring in old persons,
it will always be found on close inves-
tigation that the patient suffered
similarly in youth.
The method of treatment varies ac-
cording to the stage of the disease, and
is determined by the duration, the state
of the articular surfaces, the position of
the affected limb, and the general con-
stitutional condition of the sufferer.
In an ordinary case iu which the onset
cannot be clearly traced to an injury,
the first sign observed is that the patient
has a slight limp or awkwardness in his
gait. If questioned, he may admit that
he has some pain or uneasiness in the
joint, but at this stage he complains
Jittle of it. As time goes on, however,
the lameness becomes more evident, and
the pain increases, becoming localized
in one Bpot, so that the patient en-
deavours to save the limb as much
as possible by keeping it at rest and
rigid.
1 Examination, say a month or six weeks after the
patient first complains, shows that the limb on the
affected side is, as a whole, smaller than its fellow, but
that the knee appears to be much the larger. .The
muscles on the diseased side bave undergone consider-
able atrophy, and this, together with some swelling 01
the joint, gives these appearances. The swelling has
the horse-shoe shape of the synovial cavity, and jAja
obscures the normal bony prominences, especially |||||
the patella. To the touch it has a peculiar soft, ?
doughy, or boggy feel-
ing, and the temperature
is slightly raised. Pain may
be elicited by twisting or per-
cussing the joint in any way
which brings the articular
surfaces in contact.
"When the joint is in this state the treatment consists
in general tonics, with pressure, absorbents and rest
locally. The general hygiene must be made as
nearly perfect as possible. Good food, open-air exer-
cise, cod liver oil, and, if necessary, some such mixture
as the following : R. Syr. Ferri iodidi, Syr. Ferri phos.
co., Syr. Calcis lactophos. di 31. misce. Sig. A tea-
spoonful thrice daily after food.
The pressure and absorbents are conveniently com-
bined in the well-known "Scott's dressing." Before
applying this dressing the limb is thoroughly cleansed
with soap and water. A thick layer of strong mercurial
ointment (unguentum hydrargyri compositum, B.P.)
is spread on a sheet of lint, so shaped as accurately to
cover the joint and the limb for an inch or two above
and below it. This is fixed, and firm pressure applied
t>y a series of strips of strong moleskin plaster, about
half an inch broad, and long enough to extend one and
a quarter times round the joint. These are applied from
below upwards, each one overlapping two-thirds of its
predecessor till the whole joint is covered in. A layer
of wool and an elastic bandage from the toes upwards
complete the dressing. A Thomas s knee splint, or a
plaster of Paris case, gives the necessary fixity to the
joint, without preventing the patient taking outdoor
exercise. The Scott's dressing becomes loose in a fort-
night or three weeks, and must be reapplied. Thomas's
knee splint (Fig. 1) consists of two paral el i-on bars
surmounted by an oval, padded, metal rim?, which is
formed to fit into the perineum on the affected side.
The lower end is also an oval ring, which rests on the
ground beyond the level of the foot. In tbis way the
patient really sits on the upper end, with the diseased
leg hanging in the splint, to which it is bandaged above
and below the knee, a brace passing over the opposite
shoulder. A patten (Fig. 2) is fixed to the boot of the
opposite foot to equalise the length of the limbs, and
the patient walks with the aid of crutches.
A further stage of the disease is marked by advanced
afrophy of the muscles, with great swelling of the joint,
which becomes globular, tense, and shining, with large
superficial veins on the surface, and often un excessive
growth of hair round about it. The pain on movement
is increased, and the patient complains of occasional
sudden jerking pains in the joint occurring just as heis
dropping off to sleep.
In addition to the general treatment mentioned
above, absolute rest in bed is indicated. This is beat
accomplished by applying extension with weight and
pulley to the affected leg, or, in a restless child, by ex-
tension to both legs. The legs are thoroughly cleansed
and enveloped in a boracic lint bandage, over which the
extension plasters are applied. Thene consist of pieces
of strong moleskin plaster (Fig. 3) cut tr? the shape of
the limb, extending from the middle of the thigh to
beyond the sole of the foot. To the lower end is sewn
a piece of strong tape, while the upper end is split
longitudinally into three tails, which are folded down
and incorporated with the linen bandage which fixes
Fig. 3.
ROYAL INFIRMARY, EDINBURGH. is spread on a sheet of lint, so shaped as accurately to
The Treatment oe The?* Dxsease oe the ? wT't^s JffiSS
Knee Joint. by
a series of strips of strong moleskin plaster, about
It is now generally admitted that the condition half an inch broad, and long enough to extend one and
i , ... ir a quarter times round the joint. 1 hese are applied from
known as gelatinous degeneration, or white swelling b(^ow rda> each one overlapping two-tbirds of its
mg of the knee, is tubercular m its nature. It occurs predecessor till the whole joint is covered in. A layer
almost invariably in the young, and may begin as an 0f wool and an elastic bandage from the toes upwards
acute inflammation set up by direct injury, exposure to complete the dressing. A Thomas s knee splint, or a
cold or damp, or by some other dis-
tinctly traceable cause; or, on the other
band, the onset may be slow and in-
sidious, when the only evident cause
discoverable is the strumous diathesis of
the patient, which predisposes him to . _ . , ,,
such affections. In the rare cases of plaster of Paris case, gives the necessary hxity to the
white swelling occurring in old persons, joint,^ without preventing the patient taking^ ou ooi
it will always be found on close inves- exercise. The Scott s dressing becomes loose in a or^-
tigation that the patient suffered night or three weeks, and must be reapplied. Thomas s
similarly in youth. knee splint (Fig. 1) consists of two paral el i-on bars
The method of treatment varies ac- surmounted by an oval, padded, metal rin^, which is
cording to the stage of the disease, and formed to fit into the perineum on the affected side,
is determined by the duration, the state The lower end is also an oval ring, which rests on the
of the articular surfaces, the position of ground beyond the level of the foot. In tttis way the
the affected limb, and the general con- patient really sits on the upper end, with the diseased
stitutional condition of the sufferer. le8 hanging in the splint, to which it is bandaged above
In an ordinary case in which the onset and below the knee, a brace passing over the opposite
cannot be clearly traced to an injury, shoulder. A patten (Fig. 2) is fixed to the boot ot the
the first sign observed is that the patient opposite foot to equalise the^ length ot the limb?, and
has a slight limp or awkwardness in his the patient walks 'wMi the aid of crutches.
gait. If questioned, he may admit that ^ further stage of the disease is waited by advanced
he has some pain or uneasiness in the afrophy of the muscles, with great swelling of the joint,
joint, but at this stage he complains which becomes globular, tense, and shining, with large
Jittle of it. As time goes on, however, superficial veins on the surface, and often u.n excessive
the lameness becomes more evident, and growth of hair round about it. The pain on movement
the pain increases, becoming localized is increased, and the patient complains of occasional
in one spot, so that the patient en- sudden jerking pains in the joint occurring just as heis
deavours to save the limb as much dropping off to sleep. . .
as possible by keening it at rest and In addition to the general^ treatment mentioned
Fig. i. ri & above, absolute rest in bed is indicated. This is best
r? Examination, say a month or six weeks after the accomplished by applying extension with weight and
patient first complains, shows that the limb on the
affected side is, as a whole, smaller than its fellow, but
that the knee appears to be much the larger. The
muscles on the diseased side have undergone consider-
able atrophy, and this, together with some swelling of
the joint, gives these appearances. The swelling has
the horse-shoe shape of the synovial cavity, and
obscures the normal bony prominences, especially
the patella. To the touch it has a peculiar soft,
doughy, or boggy feel-
ing, and the temperature
is slightly raised. Pain may
be elicited by twisting or per-
cussing the joint in any way
p ~ which brings the articular
wu . surfaces in contact.
. "hen the joint is in this state the treatment consists
in general tonics, with pressure, absorbents and rest
locally. The general hygiene must be made as
nearly perfect as possible. Good food, open-air exer- pulley to the affected leg, or, in a restless child, by ex-
cise, cod liver oil, and, if necessary, some such mixture tension to both legs. The legs are thoroughly cleansed
as the following : R. Syr. Ferri iodidi, Syr. Ferri phos. and enveloped in a boracic lint bandage, over which the
co., Syr. Calcis lactophos. da gi. misce. Sig. A tea- extension plasters are applied. The^e consist of pieces
spoonful thrice daily after food. of strong moleskin plaster (Fig. 3) cut tr> the shape of
The pressure and absorbents are conveniently com- the limb, extending from the middle of the thigh to
bined in the well-known "Scott's dressing." Before beyond the sole of the foot. To the lower end is sewn
applying this dressing the limb is thoroughly cleansed a piece of strong tape, while the upper end is split
with soap and water. A thick layer of strong mercurial longitudinally into three tails, which are folded down
ointment (unguentum hydrargyri compositum, B.P.) and incorporated with the linen bandage which fixes
138 THE HOSPITAL, Nov. 26, 1892.
on the plaster. After the plaster has firmly adhered
(six to twelve hours) the weights, from four to eight
pounds, according to circumstances, may be applied,
and hung over the end of the bed on some appliance
which will carry them clear of the bedclothes and
frame. (Fig. 4),
The extension apparatus must be changed every few
weeks, lest the skin be irritated by the plaster or con-
stricted by the bandage.
In a still later stage of the disease the ligaments of
the joint soften and become useless, permitting dis-
location to take place. Abscesses form in and around
the articulation, the pain is severe, preventing sleep at
night, and the patient rapidly goes down hill from ex-
haustion, waxy disease and hectic. It is to prevent
this unfortunate result that the operation of excision is
performed when rest with extension fails to cure the
condition. The aim in this operation is to remove all
the diseased tissues in the region of the joint, namely,
the synovial membrane, articular surfaces of the bones,
and some of the ligaments. A semilunar flap is cut by
an incision pasBing from the back of one condyle to
the back of the other, crossing the front of the limb at
the level of the head of the tibia. This is dissected
up well above the patella and the tendon of the
quadriceps muscle, with the fibres of the vasti, are
carefully cut through. In this way the diseased and
swollen synovial membrane is exposed, and can with
care be dissected out entire. Its attachments to the
articular surface of the femur is cut; the patella lies
embedded in the mass and is removed with it. The
ligaments are all scraped thoroughly with a Lister's
sharp spoon, until they are white and shining, then
they are cut. The articular surfaces of the bones are
sawn off, and the adjacent ends fixed together with
steel skewers. An antiseptic dressing is applied, and
the limb fixed up in a Watson's splint with plaster
of Paris.
Should excision fail to remove all disease, nothing
remains but to amputate the limb above knee.

				

## Figures and Tables

**Fig. 1. Fig. 2. Fig. 3. Fig. 4. f1:**